# Maternal effects mediated by egg quality in the Yellow-legged Gull *Larus michahellis *in relation to laying order and embryo sex

**DOI:** 10.1186/1742-9994-8-24

**Published:** 2011-10-19

**Authors:** Diego Rubolini, Maria Romano, Kristen J Navara, Filiz Karadas, Roberto Ambrosini, Manuela Caprioli, Nicola Saino

**Affiliations:** 1 Dipartimento di Biologia, Università degli Studi di Milano, Via Celoria 26, I-20133, Milano, Italy; 2 Department of Poultry Science, University of Georgia, 203 Poultry Science Building, Athens, GA 30602, USA; 3 University of Yüzüncü Yýl, Faculty of Agriculture, Department of Animal Science, 65080 Van, Turkey; 4 Dipartimento di Biotecnologie e Bioscienze, Università degli Studi di Milano-Bicocca, P.zza della Scienza 2, 20126 Milano, Italy

**Keywords:** androgens, birds, antioxidants, carotenoids, corticosterone, egg size, estradiol, hormones, laying order, testosterone

## Abstract

**Background:**

Maternal effects mediated by egg size and quality may profoundly affect offspring development and performance, and mothers may adjust egg traits according to environmental or social influences. In avian species, context-dependency of maternal effects may result in variation in egg composition, as well as in differential patterns of covariation among selected egg components, according to, for example, position in the laying sequence or offspring sex. We investigated variation in major classes of egg yolk components (carotenoids, vitamins and steroid hormones) in relation to egg size, position in the laying sequence and embryo sex in clutches of the Yellow-legged Gull (*Larus michahellis*). We also investigated their covariation, to highlight mutual adjustments, maternal constraints or trade-offs in egg allocation.

**Results:**

Laying sequence-specific patterns of allocation emerged: concentration of carotenoids and vitamin E decreased, while concentrations of androgens increased. Vitamin A, estradiol and corticosterone did not show any change. There was no evidence of sex-specific allocation or covariation of yolk components. Concentrations of carotenoids and vitamins were positively correlated. Egg mass decreased along the laying sequence, and this decrease was negatively correlated with the mean concentrations of carotenoids in clutches, suggesting that nutritionally constrained females lay low quality clutches in terms of carotenoid content. Finally, clutches with smaller decline in antioxidants between first- and last-laid eggs had a larger increase in yolk corticosterone, suggesting that a smaller antioxidant depletion along the laying sequence may entail a cost for laying females in terms of increased stress levels.

**Conclusions:**

Since some of the analyzed yolk components (e.g. testosterone and lutein) are known to exert sex-specific phenotypic effects on the progeny in this species, the lack of sex-specific egg allocation by mothers may either result from trade-offs between contrasting effects of different egg components on male and female offspring, or indicate that sex-specific traits are controlled primarily by mechanisms of sexual differentiation, including endogenous hormone production or metabolism of exogenous antioxidants, during embryonic development.

## Background

In oviparous species, egg size and composition are important components of offspring fitness, with short-term consequences on pre- and early post-natal life-stages as well as enduring effects into adulthood [1-3]. While egg size and composition can be considered as maternal phenotypic traits, the fitness consequences of their variation are typically expressed in the offspring, and these traits are therefore expected to mediate important epigenetic maternal effects [1,2]. Such effects may contribute to adaptively modulate phenotypic variation among progeny members in relation to ecological conditions [4-6]. Epigenetic influences on the offspring evolve under the constraints set by maternal physiology and ecological conditions [3,7]. Importantly, since different egg components may have integrated effects on the offspring, natural selection is expected to operate on the combination of maternal effects via the egg and to promote a balance among egg components [3,8,9].

Previous studies of birds have analysed the sources of variation in the concentration of several egg components (e.g. carotenoids and steroid hormones), such as ecological conditions, progression of the season or parental phenotype [e.g. [3,8,10-13]]. Evidence has also been provided for a covariation between egg components and offspring sex at very early stages of egg incubation and embryo development [e.g. [9,14-16]], although this was not the case in many other studies [e.g. [8,15,17-19]]. Importantly, there is a dearth of studies where the covariation among egg components has been investigated [8,20], and even more sparse are the studies where the variation in the patterns of association among different classes of egg components has been analysed in relation to position in the laying sequence or sex of the zygote [9], which can greatly impact on the reproductive value of the offspring [8,9]. Yet, differential patterns of covariation may be expected for diverse reasons. For example, limitation of dietary resources (such as vitamins) or costs of transfer of endogenous maternal substances (such as hormones) may vary among egg components. Maternal allocation strategies may therefore entail differential allocation of some egg components depending on the reproductive value of individual offspring as influenced by position in the laying sequence and, hence, in the within-brood size/age hierarchy, and by sex [8,9,21]. Even in the absence of strategic maternal allocation to the eggs, physiological constraints or depletion of maternal resources with, for example, laying of consecutive eggs can alter the observed covariation among egg components. Moreover, offspring of either sex may differ in physiological requirements and susceptibility to individual egg components [e.g. [22-26]], thus paving the way for the evolution of allocation of different amounts of 'ingredients' to eggs of either sex. Finally, mechanisms of sex determination may themselves contribute to affect the relative content of individual components in eggs carrying either sex [e.g. [27,28]].

Therefore, not only variation in concentration of individual egg components should be expected to occur according to e.g. laying order or embryo sex, but also variation in the patterns of association among egg components should occur as a result of maternal strategies, constraints, and/or ecological effects. Describing the patterns of covariation among egg components is therefore pivotal to understand the evolution of maternal effects and the impact of ecological conditions on reproductive strategies of oviparous species.

Vitamin E and carotenoids transferred to egg yolks are expected to provide protection against oxidative damage arising during rapid embryonic growth, and may further act as immunomodulators, immunostimulators and regulators of cell differentiation and proliferation [29-31]. Though the role of carotenoids as antioxidants in avian species is currently debated [e.g. [32]], there is experimental evidence that vitamin E can reduce oxidative damage and positively affect offspring growth and performance [33]. Carotenoids and vitamin E are of dietary origin, as birds cannot synthesize these substances *de novo *[29,34]. Moreover, vitamin A (retinol), though it has weak antioxidant properties, is a precursor of retinoic acid, exerts important systemic functions in embryo development, and is involved in cell differentiation, as well as neuronal plasticity [35,36]. Vitamin A can be synthesized via carotenoid conversion, especially from carotenes [29].

Egg androgens have both activational and organizational functions, by e.g. enhancing muscle development, growth and chick competitiveness in sibling rivalry, with long-lasting effects on sexual behaviour, dominance, and expression of secondary sexual traits [reviews in [3,10,37]]. On the other hand, corticosterone in eggs is probably a byproduct of maternal physiology, and stressed mothers are known to lay eggs with elevated corticosterone levels [38-40] that produce smaller and less viable chicks [26,39,41]. Both androgens and corticosteroid transmission can entail fitness costs to the chicks in terms of a reduced immune response, which may negatively affect the ability to fend off parasites [41,42]. The possible functions of estradiol in egg yolk are still unclear, as its phenotypic effects have never been investigated under natural conditions [43,44].

The few studies investigating covariation among concentrations of different egg components highlighted that two major classes of egg biochemical constituents, antioxidants and hormones, show idiosyncratic variation in their patterns of association among species [8,9,45], whereas they have documented a positive covariation between different carotenoids, and between carotenoids and vitamin E [20]. Moreover, differences among years in patterns of covariation among specific egg components (carotenoids and vitamin A) have been observed, suggesting that temporal variation in ecological conditions, such as weather conditions possibly affecting availability of carotenoid-rich food supplies, may modify the covariation among egg micronutrients [20].

In this study of the Yellow-legged Gull (*Larus michahellis*), we first focus on the analysis of the variation of major functional classes of egg components, i.e. carotenoids and vitamins (A and E), and steroid hormones (testosterone, estradiol, corticosterone) according to laying order and embryo sex. Based on previous studies of closely-related gull species [8,46], we expected androgen levels to increase along the laying sequence, and carotenoids to decrease. Since yolk testosterone is known to exert sex-specific effects in the study population, with testosterone depressing female offspring fitness more than male fitness [24], we also expected female eggs to contain less androgens than male ones, whereas carotenoids were not predicted to covary with embryo sex [22]. Moreover, males are more susceptible than females to poor rearing conditions [47], and thus maternal egg investment may be expected to vary with offspring sex according to its reproductive value and the constraints set by maternal conditions [48,49].

Secondly, we investigate the patterns of covariation among different egg components, both at the within-clutch and at the among-clutches levels, and in relation to embryo sex and laying order. We also explore potential maternal trade-offs and energetic constraints on resource allocation to reproduction by analysing whether variation in egg components among clutches vary according to total clutch investment [an index of the total amount of clutch macronutrients; e.g. [2,50]].

To our knowledge, this is one of the very few studies investigating simultaneously the sex-related variation and the covariation of carotenoids, vitamins and two important egg hormones besides egg androgens, which were the main focus of previous studies [8,9,45].

Yellow-legged Gulls lay clutches of a maximum of 3 eggs. Last laid c-eggs are laid on average 4 days after first laid a-eggs (DR, NS: unpubl. data) and are up to 20% smaller than a-eggs [see Table 1; see also [49]]. Hatching is asynchronous: chicks from c-eggs hatch on average 2 days later than their siblings from a-eggs [47], are smaller and suffer from competitive disadvantage compared to their older siblings, resulting in higher pre-fledging mortality [49,51]. Similar to closely-related species [52,53], Yellow-legged Gulls are sexually size dimorphic soon after hatching [22], with males becoming 15-20% larger than females once adult size is reached [54].

## Results

### Variation in egg traits in relation to laying order and sex

**Figure 1:**
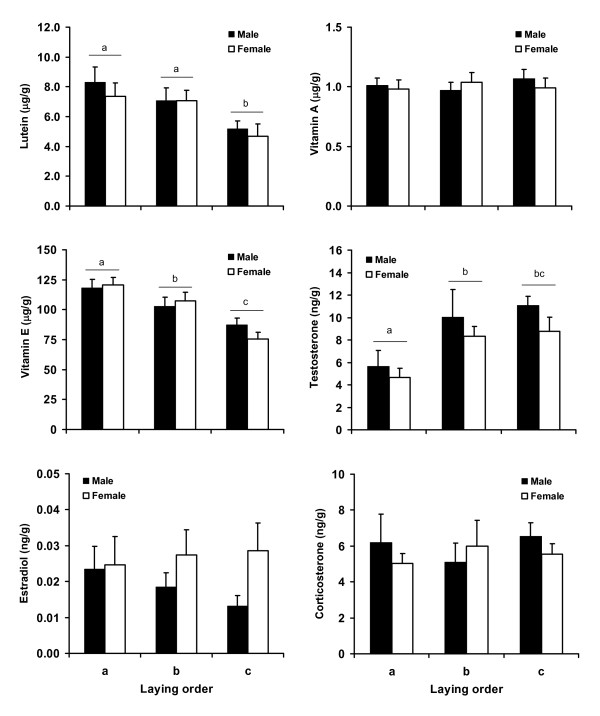
**Concentrations of different egg components in relation to laying order and sex**. Values are mean and associated standard errors. Letters denote statistically significant differences between levels of laying order at *post-hoc *tests from mixed models including sex and laying order as fixed effects, and clutch identity as a random factor (see Results).

Mixed models of all measured egg traits in relation to laying order revealed laying-sequence specific maternal transfer of components in several cases: the concentration of all carotenoids and vitamin E decreased along the laying sequence, whereas the concentration of all androgens increased (Table 1, Figure 1). The concentration of vitamin A, estradiol and corticosterone did not vary with laying order (Table 1, Figure 1).

**Table 1:** Summary statistics of egg traits in relation to laying order

	a-egg			b-egg			c-egg		
	
	Mean	**s.d**.	*n*	Mean	**s.d**.	*n*	Mean	**s.d**.	*n*
Egg mass (g)	93.1	5.0	30^a^	89.7	4.8	30^b^	84.2	5.8	30^c^
Lutein (μg/g)	7.89	3.81	29^a^	7.06	3.10	30^a^	4.94	2.57	30^b^
*cis*-lutein (μg/g) ^§^	1.05	1.13	29^a^	0.99	0.87	30^a^	0.69	0.99	30^b^
Zeaxanthin (μg/g) ^§^	4.09	3.13	29^a^	3.95	2.23	30^a^	2.91	2.50	30^b^
Dehydrolutein (μg/g) ^§^	2.71	3.13	29^a^	1.85	2.11	30^ab^	1.30	1.52	30^b^
*ß*-cryptoxanthin (μg/g) ^§^	1.07	0.70	29^a^	0.87	0.63	30^a^	0.52	0.47	30^b^
*ß*-carotene (μg/g)	3.56	1.69	29^a^	3.07	2.00	30^ab^	2.78	1.56	30^b^
Total carotenoids (μg/g)	22.2	10.1	29^a^	19.7	6.9	30^a^	14.6	6.6	30^b^
Vitamin A (μg/g)	1.00	0.26	30	1.00	0.28	30	1.03	0.31	30
Vitamin E (μg/g)	119.2	26.4	30^a^	104.9	28.1	30^b^	81.4	22.4	30^c^
Testosterone (ng/g) ^§^	5.26	4.93	30^a^	9.28	7.83	30^b^	9.94	4.04	30^bc^
Androstenedione (ng/g) ^§^	101.2	46.9	21^a^	165.0	92.7	20^b^	213.6	103.4	20^bc^
Dihydrotestosterone (ng/g)	1.66	1.00	9^a^	2.13	0.75	9^a^	4.76	2.00	9^b^
Estradiol (ng/g) ^§^	0.024	0.026	30	0.022	0.021	30	0.021	0.024	30
Corticosterone (ng/g) ^§^	5.70	4.87	28	5.46	4.43	27	6.02	2.52	29

Different superscript letters denote statistically significant (*P *< 0.05) differences in *post-hoc *tests from mixed models with clutch identity as a random factor and laying order as a fixed effect (see Methods). §: statistical tests performed on log_10_-transformed values

We then ran mixed models testing the independent and combined effects of embryo sex and laying order on the concentration of different egg components (models were not run for dihydrotestosterone and androstenedione; see Methods). There were no sex differences in egg size or composition (effect of sex, all *P *> 0.10), and no sex × laying order effects (all *P *> 0.14) (Figure 1; see details of non-significant sex and sex × laying order effects in Table 2).

**Table 2:** Summary of mixed models of egg size and composition testing sex- and laying sequence-specific variability

	Laying order	Sex	Laying order × sex
Egg mass	*F*_2,57.1 _= 82.4	*F*_1,63.9 _= 2.43	*F*_2,6.3 _= 1.82
	*P *< 0.001	*P *= 0.12	*P *= 0.17
Lutein	*F*_2,56.4 _= 13.8	*F*_1, 72.1 _= 0.24	*F*_2,65.9 _= 0.04
	*P *< 0.001	*P *= 0.62	*P *= 0.96
*cis*-lutein	*F*_1,56.2 _= 7.37	*F*_1,66.7 _= 0.05	*F*_2,61.6 _= 0.73
	*P *= 0.001	*P *= 0.83	*P *= 0.49
Zeaxanthin	*F*_2,56.3 _= 7.61	*F*_1,72 _= 0.68	*F*_2,65.3 _= 0.78
	*P *= 0.001	*P *= 0.41	*P *= 0.46
Dehydrolutein	*F*_2,56.6 _= 5.55	*F*_1,72.9 _= 0.76	*F*_2,66.1 _= 1.29
	*P *= 0.006	*P *= 0.39	*P *= 0.28
*ß*-cryptoxanthin	*F*_2,56.9 _= 9.25	*F*_1,79.1 _= 0.03	*F*_2,71.5 _= 0.27
	*P *< 0.001	*P *= 0.87	*P *= 0.77
*ß*-carotene	*F*_2,56.5 _= 2.53	*F*_1,76.6 _= 0.29	*F*_2,69.5 _= 0.28
	*P *= 0.09	*P *= 0.59	*P *= 0.77
Total carotenoids	*F*_2,56.4 _= 11.84	*F*_1,75.9 _= 1.17	*F*_2,69 _= 0.09
	*P *< 0.001	*P *= 0.28	*P *= 0.91
Vitamin A	*F*_2,57 _= 0.32	*F*_1,63.3 _= 0.95	*F*_2,59.7 _= 2.01
	*P *= 0.72	*P *= 0.33	*P *= 0.14
Vitamin E	*F*_2,57.7 _= 6.79	*F*_1,85.9 _= 0.09	*F*_2,82.6 _= 0.90
	*P *< 0.001	*P *= 0.76	*P *= 0.41
Testosterone	*F*_2,57.6 _= 15.50	*F*_1,84.2 _= 1.11	*F*_2,79.1 _= 0.89
	*P *< 0.001	*P *= 0.29	*P *= 0.42
Estradiol	*F*_2,57.4 _= 0.29	*F*_1,80.5 _= 2.64	*F*_2,75 _= 0.27
	*P *= 0.75	*P *= 0.11	*P *= 0.76
Corticosterone	*F*_2,80 _= 1.38	*F*_1,80 _= 0.01	*F*_2,78 _= 0.48
	*P *= 0.26	*P *= 0.93	*P *= 0.62

Statistics for laying order and sex refer to models were the interaction term was removed.

Embryos of a-, b-, and c-eggs differed in developmental stage at the time of collection (mixed model of embryo size where laying order, sex, their interaction, and egg mass were included as fixed effects; *F*_2,59.6 _= 9.02, *P *< 0.001), b-eggs showing more developed embryos than either a- or c-eggs (*P *< 0.008 at *post-hoc *tests). There was no sex difference in embryo size (*F*_1,58.0 _= 1.17, *P *= 0.28). The sex × laying order interaction was also not significant, nor was the the additional effect of egg size (both *P *> 0.21). Therefore, our egg sampling protocol (see Methods) was not sufficient to account for differences in incubation patterns among eggs. However, all results concerning variation in egg composition in relation to laying order and sex were qualitatively unaltered when the analyses were repeated for the subset of eggs for which embryo size was measured by also including embryo size (as well as its two- and three-ways interactions with sex and laying order) in the models reported in Table 2 (details not shown for brevity).

Thus, we documented laying-sequence specific patterns of variation of yolk components, but no evidence of sex-specific effects. Importantly, differences in developmental stage of eggs according to laying order, as indexed by variation in embryo size, did not affect our conclusions.

### Predictors of among-clutch variation in egg size and composition

We explored whether among-clutches effects could explain variation in egg size and composition (except dihydrotestosterone and androstenedione; see Methods) using mixed models where we included laying date of the first egg in a clutch, number of males in a clutch, and total clutch mass (the latter variable was not tested for egg size), as well as their two- and three-ways interactions, as predictors. Models were run first by including main effects only, and interactions were then added all at the same time. None of these variables or interactions significantly predicted egg size or composition (all *P *> 0.10, details not shown for brevity), with the exception of total clutch mass, that negatively predicted estradiol levels [estimate: -3.10 × 10^-4 ^(0.76 × 10^-4 ^s.e.), *F*_1,26 _= 16.8, *P *< 0.001]. There was a significant two-way interaction between the number of males and laying date on egg mass [estimate: 0.31 (0.08 s.e.), *F*_1,26 _= 16.9, *P *< 0.001]. This latter finding indicates that females laying all-male clutches also lay larger eggs at the end but not at the beginning of the breeding season, a pattern that was already discussed in a previous analysis of the same dataset [49].

### Covariation between egg composition and egg mass: separating within- from among-clutches effects

We investigated whether main egg components (only lutein, total carotenoids, vitamin A, vitamin E, testosterone, estradiol, and corticosterone were considered in all subsequent analyses; see Methods) covaried with egg mass at the among- or within-clutch level. We ran mixed models with clutch identity as a random effect, sex, laying order and their interaction as fixed factors, and the mean within-clutch egg mass (representing an among-clutches effect), as well as the deviation of mass of individual eggs from the within-clutch mean (representing a within-clutch effect), as covariates [55]. Only clutches with complete data for each component were included in the analyses.

Egg components did not covary with egg mass, either at the within- or among-clutch levels, with the exception of estradiol, that negatively covaried with egg mass at the among-clutch level [-9.00 × 10^-4 ^(2.25 × 10^-4 ^s.e.), *F*_1,27.7 _= 16.1, *P *< 0.001] (see also previous paragraph), but not at the within-clutch level [-2.00 × 10^-4 ^(4.29 × 10^-4 ^s.e.), *F*_1,56.4 _= 0.22, *P *= 0.64]. However, though the among-clutch effect was stronger, the among- and within-clutch effects did not differ significantly [-7.00 × 10^-4 ^(4.78 × 10^-4 ^s.e.), *F*_1,77.9 _= 2.14, *P *= 0.15] [55].

### Covariation among egg components

At the within-clutch level, we found statistically significant positive correlations between concentrations of vitamin A, vitamin E and carotenoids (Table 3). Similar results were observed at the among-clutch level (Table 3). However, the strong positive correlation between carotenoids and vitamin E observed within-clutches was not found at the among-clutch level, and the within-clutch correlations were significantly stronger than the among-clutch correlations (Table 3). On the other hand, for none of the hormones did concentrations significantly correlate with the other egg components, either at the among- or within-clutch level (Table 3). The analyses were replicated for each sex separately, in a simpler multiple response mixed model with laying order as a fixed factor to investigate sex differences in the correlation coefficients between components at the two levels of variation. No correlation coefficients significantly differed between the sexes, either at the among- or within-clutch levels (all *P *> 0.13, detail not shown), with the single exception of the among-clutch relationship between vitamin A and corticosterone, that was positive and statistically significant in males (*r *= 0.49, d.f. = 25, *P *= 0.009) but weakly negative and nonsignificant in females (*r *= -0.08, d.f. = 21, *P *= 0.71) (test of the difference between two correlation coefficients, *z *= 2.03, *P *= 0.042).

**Table 3:** Correlation matrix between egg components

*Among-clutches correlations*					
	Vitamin A	Vitamin E	Testosterone	Estradiol	Corticosterone
	
Lutein	0.56*	*0.13*	-0.21	0.01	-0.05
Total carotenoids	0.63*	*-0.01*	0.01	-0.09	-0.01
Vitamin A		0.42*	0.10	-0.28	0.27
Vitamin E			-0.06	0.29	0.14
Testosterone				0.07	-0.02
Estradiol					0.32
*Within-clutch correlations*					
	Vitamin A	Vitamin E	Testosterone	Estradiol	Corticosterone
	
Lutein	0.40*	0.61*	-0.07	0.02	0.12
Total carotenoids	0.40*	0.71*	0.01	-0.01	0.16
Vitamin A		0.58*	-0.04	0.06	0.14
Vitamin E			-0.01	0.12	0.30
Testosterone				0.08	0.23
Estradiol					-0.04

Correlation coefficients were derived from multiple response mixed models while accounting for laying order, embryo sex and their interaction (see Methods and Results). Significance of correlation coefficients is calculated based on a conservative sample size of 30 (equal to the number of clutches included in the comparisons). Asterisks denote correlations with *P *< 0.05, while values in *italics *indicate correlation coefficients that significantly differed (*P *< 0.05) at the among- and within-clutch levels.

We then investigated the correlation between egg components within each level of laying order (Table 4), which broadly confirmed the correlation patterns disclosed by previous analyses (see Table 3). In addition, in a-eggs only, we found negative correlations between estradiol and vitamin A or vitamin E, as well as a positive correlation between corticosterone and vitamin E (Table 4). Pairwise comparisons of correlation coefficients revealed 4 statistically significant differences in correlation patterns between a- and c-eggs, 2 differences between a- and b-eggs, and no differences between b- and c-eggs (see Table 4). Specifically, the negative relationships between both vitamins and estradiol were not found in b- and c-eggs, whereas lutein negatively covaried with estradiol in a-eggs but positively in c-eggs, and the positive relationship between vitamins A and E was stronger among a-eggs than c-eggs (Table 4).

**Table 4:** Correlation matrix between egg components for each level of laying order.

	**Vitamin A**	**Vitamin E**	**Testosterone**	**Estradiol**	**Corticosterone**
	
*a-eggs* Lutein	0.52*	0.44*	-0.06	*-0.25*^c^	0.06
Total carotenoids	0.58*	0.46*	0.07	-0.30	0.18
Vitamin A		0.70*^c^	0.13	-0.58*^b,c^	0.32
Vitamin E			0.06	-0.59*^b,c^	0.43*
Testosterone				0.22	0.21
Estradiol					0.04
* b-eggs*					
Lutein	0.57*	0.46*	-0.35	0.11	-0.03
Total carotenoids	0.68*	0.57*	-0.12	0.15	0.01
Vitamin A		0.55*	0.03	*0.04*^a^	0.13
Vitamin E			-0.17	*0.28*^a^	0.23
Testosterone				0.13	0.21
Estradiol					0.28
*c-eggs*					
Lutein	0.53*	0.45*	0.07	*0.31*^a^	0.25
Total carotenoids	0.50*	0.48*	0.17	0.07	0.22
Vitamin A		*0.21*^a^	0.00	*0.14*^a^	0.22
Vitamin E			0.13	*-0.07*^a^	0.28
Testosterone				-0.22	0.13
Estradiol					-0.08

Sample size varies between 27 and 30 eggs, depending on the specific comparison. Asterisks denote correlations with *P *< 0.05, while letters and *italics *indicate correlation coefficients that significantly differ (*P *< 0.05) in pairwise comparisons between levels of laying order [a specific letter (a, b, or c) indicates the level of laying order to which the comparison refers].

To summarize, mothers that laid clutches or eggs with more vitamin A also laid clutches or eggs with more vitamin E and carotenoids, while, within clutches, eggs with larger concentration of vitamin E had also higher concentrations of carotenoids, and there were weak sex differences in the correlation between vitamin A and corticosterone only. Correlations within each level of laying order were broadly similar, though there were some differences, mainly between a- and c-eggs.

### Covariation among gradients of egg components along the laying sequence and egg mass asymmetry

**Figure 2:**
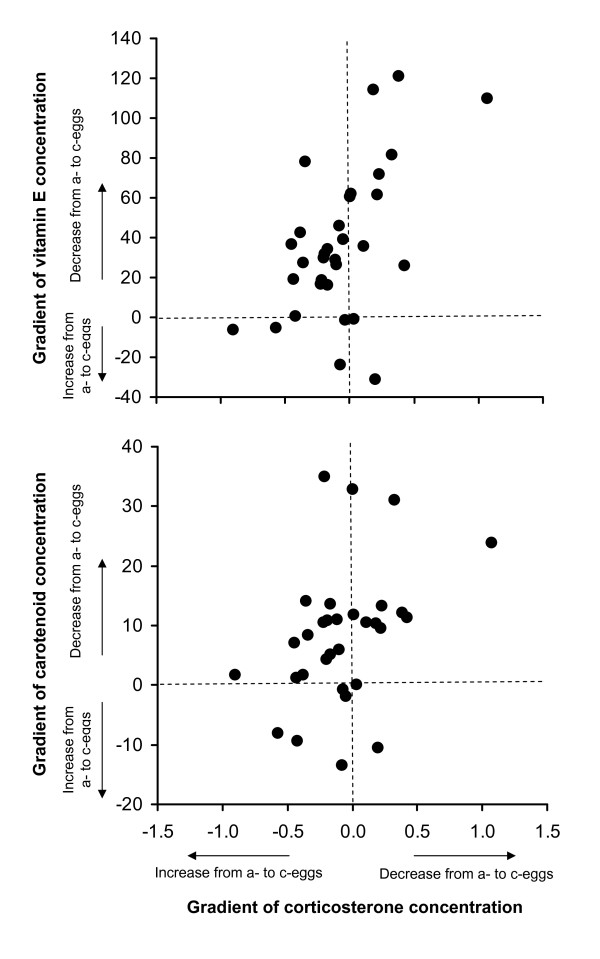
**Relationships between gradient of vitamin E or total carotenoid concentrations and gradient in corticosterone concentration**. Gradients along the laying sequence are expressed as (a- minus c-eggs); gradient in corticosterone concentrations was calculated based on log_10_-transformed values (see Methods).

Correlation coefficients between within-clutch gradients of egg components along the laying sequence (expressed as differences in egg components between a- and c-eggs for each clutch) were positive and statistically significant in several cases involving vitamin E, vitamin A and carotenoids (vitamin A and vitamin E, vitamin E and lutein, vitamin E and total carotenoids, *r *> 0.55 in all cases), paralleling positive correlations highlighted in previous analyses at the within-clutch level (see Table 3). In addition, we found a positive correlation between gradients of vitamin E or total carotenoids and gradients of corticosterone (*r *= 0.55, *n *= 27, *P *= 0.003 and *r *= 0.41, *n *= 26, *P *= 0.037, respectively) (Figure 2), and a negative correlation between gradients of vitamin E and gradients of estradiol (*r *= -0.40, *n *= 30, *P *= 0.028). All other correlations among gradients in egg components between a- and c-eggs were not significant (all *P*-values > 0.10, details not shown for brevity).

**Figure 3:**
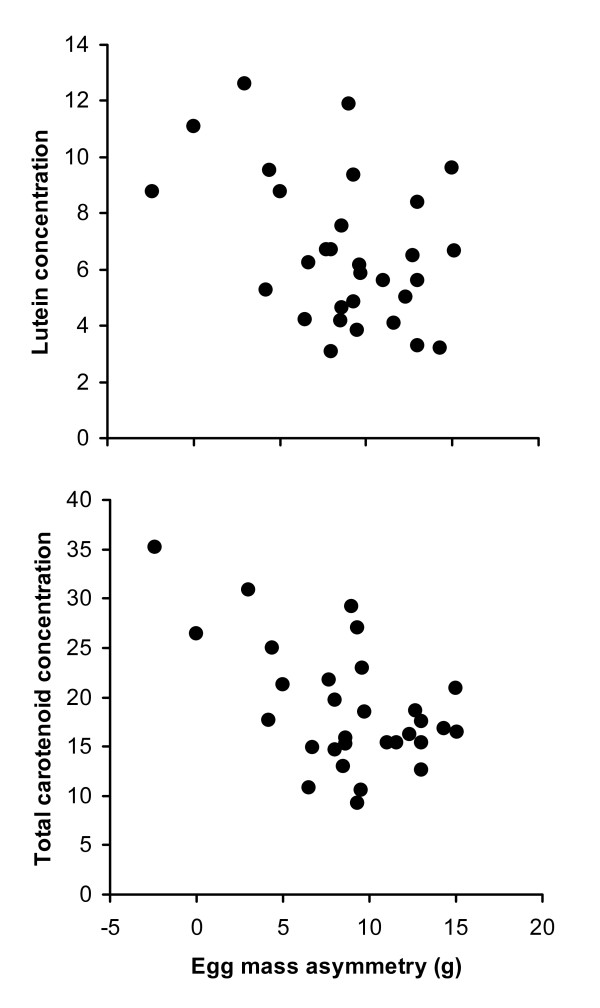
**Relationships between mean within-clutch lutein (upper) or total carotenoid (lower) concentrations and egg mass asymmetry**. Egg mass asymmetry is the difference in mass between a- and c-eggs.

We then analysed whether gradients in egg mass along the laying sequence (egg mass asymmetry; mass of a-eggs minus mass of c-eggs) predicted mean egg composition at the clutch level, or the gradients of egg components along the laying sequence. Egg mass asymmetry can be regarded as an index of nutritional constraints of laying females, as females laying similar a- and c-eggs should be able to sustain equal resource allocation among all eggs [possibly reducing chick competitive asymmetries within clutches; e.g. [56]], whereas females laying small c-eggs are likely of lower phenotypic quality and constrained in allocating energetic resources to reproduction [e.g. [57-60]]. Egg mass asymmetry negatively covaried with mean lutein (*r *= -0.41, *n *= 30, *P *= 0.023) and mean total carotenoid concentrations (*r *= -0.54, *n *= 30, *P *= 0.002) (Figure 3). These relationships were not affected by controlling for mean egg mass of clutches or mass of a-eggs in partial correlation analyses (*r *≤ -0.40 and *r *≤ -0.52, respectively, all *P *≤ 0.032), because egg mass asymmetry is only weakly negatively related to mean egg mass (*r *= -0.19, *P *= 0.31) and weakly positively related to size of a-eggs (*r *= 0.22, *P *= 0.24). All other relationships between egg mass asymmetry and mean concentrations of vitamins or hormones were not statistically significant (all |*r*| < 0.31, *P *> 0.09). On the other hand, gradients of each component along the laying sequence did not correlate significantly with egg mass asymmetry (all |*r*| < 0.28, *P *> 0.14).

## Discussion

In this study of Yellow-legged Gulls we investigated variation of egg composition (in terms of vitamins, carotenoids, and steroid hormones) in relation to laying sequence and embryo sex, as well as covariation among different egg components at the within- and among-clutch levels. The main aim of our study was to highlight patterns of maternal egg resource transfer that may be interpreted in terms of maternal effects. We also discuss, mainly from a maternal perspective, the possible causes and consequences of some of the observed associations between egg traits.

### Variation in egg composition in relation to laying sequence

We found well-defined patterns of variation of vitamins, carotenoids and hormones along the laying sequence. Specifically, the concentration of vitamin E and of carotenoids decreased, while the concentration of androgens increased along the laying sequence. On the other hand, concentrations of vitamin A, estradiol and corticosterone did not vary with laying order. Although eggs of different positions within the laying sequence were naturally incubated for different periods of time to allow for embryo development, thus potentially confounding laying-sequence specific patterns of egg androgen variation [61], we took two precautions to evaluate the possible impact of our sampling protocol on the outcome of the analyses: first, we compared mean hormone levels of a sample of unincubated clutches with those of incubated ones, and found no difference in mean yolk hormone levels (see Methods); secondly, we included a proxy of embryo development as a covariate in our statistical models, and found no statistical effects of its inclusion. We are therefore confident that our findings are not biased by laying-sequence specific patterns of incubation.

The results concerning vitamin E, carotenoids and androgens are in line with previous findings on closely-related gull species (*L. fuscus *and *L. ridibundus*) [[8,46,62,63]; see also [11]]. In these species, as well as in the Yellow-legged Gull, last laid c-eggs are typically smaller and almost invariably hatch later than previous ones, originating chicks that suffer from a severe competitive handicap compared to their older siblings, and with a lower reproductive value [e.g. [8,46,47,51,57]]. Thus, mothers appear on one hand to increase the fitness disadvantage of c-chicks by allocating less vitamins and carotenoids, but on the other they may increase their competitive ability by allocating more androgens to last-laid eggs [8,46]. An increase of egg androgen allocation along the laying sequence is far from being an universal pattern in birds [e.g. [3]], and may even be reversed in some species, like egrets, whereby parents may boost the competitive advantage of early hatched chicks and promote sibling aggression and facultative brood reduction [64]. Interspecific variation in patterns of egg androgen allocation along the laying sequence may thus reflect differences in reproductive strategies among species or populations (in terms of 'brood survival' vs. 'brood reduction') [*sensu *[65]] and contribute to either mitigate or exacerbate the consequences of asynchronous hatching of eggs [3].

The lack of laying sequence-specific variation in vitamin A concentrations is also consistent with previous findings, suggesting that dietary vitamin A may not be limiting under natural conditions, or that this egg component may not be involved in maternal strategic egg allocation [46,62]. On the other hand, the lack of variation in estradiol and corticosterone according to laying order is difficult to interpret, owing to the paucity of studies dealing with laying sequence patterns of variation and fitness effects of these hormones (see Introduction). A previous study on starlings (*Sturnus vulgaris*) found an increase of yolk corticosterone levels along the laying sequence, that would be consistent with a decrease in egg quality of last-laid eggs [66]. In fact, corticosterone in eggs may be a by-product of maternal stress response and may lead to lower quality offspring [e.g. [38,39,41]]. Previous studies investigating within-clutch variation of estradiol levels in different bird species did not reveal consistent patterns related to laying sequence, either reporting a decrease or no variation [67-69], or even sex-specific variation with laying order [9].

### Variation in egg composition in relation to embryo sex

No sex differences in mean egg composition were found, and the covariation among different egg components did not vary markedly in relation to offspring sex: out of 40 pairwise comparisons, only a single correlation (between egg corticosterone and vitamin A) showed a negligible marginally significant difference between the sexes. Thus, evidence that females packed their eggs differently for offspring of either sex was weak at best. In previous studies of the same species, high yolk testosterone reduced female viability [24], and high yolk lutein had differential effects on offspring of either sex in relation to hatch order, promoting growth of sons from a-eggs but depressing that of sons from c-eggs [22]. In addition, the effects of high yolk lutein on daughters varied in the course of the season [22]. As discussed earlier, it is possible that egg composition reflects a trade-off between the opposing effects of carotenoids and hormones on offspring phenotype in a species where no mechanisms of sex-related variation in egg composition have evolved [22]. Alternatively, the observed sex-specific effects of testosterone and lutein in spite of a lack of sex-specific maternal transfer to eggs of these substances may indicate that sex-specific features of embryonic development, including metabolism of exogenous substances (such as vitamins and carotenoids) and endogenous hormone secretion at early embryonic stages [70], play a pivotal role in sexual differentiation [see discussion in [71]].

### Covariation among egg components, and between egg components and egg mass

Vitamin E positively covaried with carotenoids within but not among clutches, while vitamin A positively covaried with carotenoids and vitamin E at both levels of variation. Thus, eggs with elevated carotenoid concentrations also had high vitamin E concentrations, but mothers allocated independent amounts of these substances to entire clutches overall. A positive covariation between carotenoids and vitamin E at the within-clutch level may suggest a similarity in the metabolic pathways that allow allocation of carotenoids and vitamin E to egg yolks [see discussion in 20], whereas a lack of covariation among clutches may depend on among-females differences in antioxidant metabolism or diet [29,34]. The positive covariation between vitamin A and carotenoids is consistent with the notion that several carotenoids [mainly carotenes, but also zeaxanthin; [72]] can be precursors of vitamin A [29]. Alternatively, it may suggest that the same dietary resources providing carotenoids and vitamin E are also rich in vitamin A.

Finally, we found no relationships between androgens and carotenoids or other substances at both the within- and between-clutches levels, with the exception of a-eggs. In fact, vitamins and carotenoids negatively covaried with yolk estradiol levels in a-eggs, but not in b- or c-eggs. Unfortunately, in light of the limited studies about the effects of yolk estradiol on progeny fitness (see Introduction), any functional interpretation of these differential patterns of covariation in relation to laying order seems premature.

The relationships between egg mass and the different egg components were generally weak or non-significant, with the exception of a strong negative relationship between clutch mass and estradiol levels. In female birds, estradiol triggers egg formation, both in terms of yolk lipid deposition and albumen synthesis [73,74]. Estradiol administration to females induces an increase in yolk precursors, vitellogenin and low-density lipoprotein, that are the primary sources of yolk proteins, although no effects of estradiol administration on egg mass or clutch size have been documented [75-77]. The observed relationship is not simply a consequence of a dilution effect of egg estradiol in large eggs. Indeed, there was a negative relationship also between total yolk estradiol and egg mass (*r *= -0.57, *P *= 0.001; yolk mass was estimated based on the regression equation between yolk mass and egg mass from a sample of freshly laid eggs; DR, NS: unpubl. data). Thus, large eggs contained less estradiol than small eggs, both in terms of per unit yolk volume and in absolute terms. This negative relationship may depend in part on differences in nutritional status of females [78], which may concomitantly affect egg estradiol deposition and egg mass in opposite ways.

Within-clutch egg size asymmetry negatively predicted the mean within-clutch carotenoid concentration. Because large egg size asymmetry may depend on poor maternal nutritional conditions [e.g. [49,59]], this results supports the "constraint hypothesis", as it suggests that female nutritional constraints affects egg carotenoid deposition [8].

Finally, we found that gradients of vitamin E and carotenoids between a- and c-eggs were positively correlated with gradients of corticosterone. The relationship between corticosterone and carotenoids or antioxidants in egg yolks has never been investigated, to our knowledge. Also in this case, functional explanations can hardly be envisaged. On the proximate side, this pattern does not derive from concomitant patterns of decrease of the two egg components along the laying sequence, since egg corticosterone did not vary with laying order. Rather, it may indicate that a reduction in carotenoid and vitamin E depletion during laying of consecutive eggs may entail a cost for laying females in terms of increased stress levels. In fact, those mothers not showing a decrease in carotenoids or vitamin E from a- to c-eggs showed an increase of egg corticosterone levels from a- to c-egg, whereas the opposite was the case for mothers showing a strong decrease of carotenoids or vitamin E along the laying sequence. The maintenance of constant levels of carotenoids or vitamin E along the laying sequence may reflect high availability of these putatively limiting substances in maternal tissues during egg laying [e.g. [29,34,79-81]], and thus reflect a greater effort by females in order to accumulate high levels of dietary antioxidants or carotenoids. Sustained high maternal corticosterone levels during egg-laying may be associated with intense food searching effort, as it is known that high basal corticosterone levels are associated with parental hyperphagia during the breeding period [82]. If the maintenance of constant carotenoid and vitamin E levels along the laying sequence, together with the concomitant change in yolk corticosterone, has positive effects on progeny fitness, females may trade their maternal stress levels against sustained allocation of carotenoids and vitamin E along the laying sequence. Obviously, this possible explanation for the observed association is entirely speculative, and rests on several assumptions, including the one that egg corticosterone levels reliably reflect maternal circulating levels [38,39].

## Conclusions

To summarize, our study confirms the existence of contrasting patterns of antioxidants and androgens variation in relation to laying order in gull clutches. However, evidence for sex-specific patterns of resource allocation to eggs was weak at best, suggesting that egg composition reflects a trade-off between contrasting effects of different egg components on offspring of either sex, or that sex-specific development is mainly controlled by sex differences in embryonic metabolism or physiology rather than by egg-mediated maternal effects. Our findings further support the notion that dietary carotenoids and vitamin E are available in limiting amounts to laying females, and that only high quality females can lay eggs with large concentrations of these substances.

## Methods

### General methods and genetic sex identification

The study was conducted at a large Yellow-legged Gull colony located in the Comacchio lagoon (N Italy) during March-June 2008. The colony was visited daily or every second day and new nests and eggs were marked to identify laying order. Only nests with a known laying order were included in the analyses. For each egg, we recorded egg mass at laying (nearest 0.1 g). We collected eggs at clutch completion according to the following protocol: a-eggs were collected on the 2^nd ^day after laying of the c-egg, b-eggs on the 3^rd ^day after laying of the c-egg, and c-eggs on the 4^th ^day after laying. The aim behind the sampling protocol was to collect all eggs in a clutch at a similar stage of embryonic development [8]. In fact, a- and b-eggs may be incubated for variable amounts of time before laying of c-eggs, and embryo development thus begins before laying of c-eggs. By adopting such a sampling protocol, we aimed at collecting all the eggs at the earliest developmental stage that allowed easy identification of the embryo for the purposes of molecular sexing, and at minimizing possible effects of slight differences in embryo developmental stage at the time of collection on egg composition [8], especially yolk hormones [[61]; but see [19,83]].

Only complete clutches with all 3 fertile eggs were included in the analyses (*n *= 30 clutches and 90 eggs). To investigate variation in egg hormones in incubated vs. unincubated eggs, an additional sample of five complete 3-egg clutches was also collected on the day of laying of each egg. Removed eggs were replaced with eggs from other nests of similar developmental stage, in order to prevent mothers' perception of egg removal, which may potentially affect egg quality or cause brood desertion. The 30 clutches included in the study were selected as representatives of the entire laying season of gulls. This was done by collecting one of every third initated clutch in the entire study colony (the proportion was based on the expected number of clutches laid in the colony in previous years). Eggs were collected under licenses issued by the relevant local (Consorzio del Parco Regionale del Delta del Po - Emilia Romagna, Prot. n. 1703, March 19^th ^2008) and national authorities (Istituto Nazionale per la Fauna Selvatica, Prot. n. 1196/TA-31, February 26^th ^2008).

Eggs were frozen at -20°C on the day of collection. Yolks and embryos were then separated and yolks stored at -80°C until biochemical analyses. We evaluated whether slight differences in embryo development at time of egg collection could influence our analyses of sex- or laying sequence-specific differences in egg composition. Stage of embryo development was estimated as the approximated size of developing yolk sac, i.e. the area covered by vitelline vessels, which is elliptical in shape. We gently removed frozen albumen and uncovered the yolk layer. However, since the boundary of the vitelline vessels pointing towards the blunt end of the egg was not clearly visible in most instances, we relied on the measurement of the minor axis (*a*) of the ellipse and of half of the major axis (*b*), from the embryo to the boundary of the vitelline sac in the direction of the acute pole of the egg. As a proxy of the stage of development, we calculated the area of developing yolk sac, estimated as [(*a *× *b*)/2] for simplicity (i.e. it was approximated to a triangle). Embryo size could be reliably recorded in a subsample of 81 eggs (out of 90 collected eggs). Embryos were then stored at -20°C until molecular sexing, which was performed based on the protocol devised by Griffiths et al. [84] and optimized for the study species. Further details about the sexing procedure can be found in Rubolini et al. [24]. We assigned the sex of all collected embryos (50 males and 40 females).

### Assay of yolk vitamins and carotenoids

The extraction of egg yolk followed established protocols [85]. In brief, egg samples (100-200 mg) were homogenized in a mixture of 0.7 ml NaCl solution (5%, w/v) and 1 ml ethanol, followed by an addition of 2 ml hexane and further homogenization. The hexane layer was collected and the hexane extraction repeated twice. The combined hexane extracts were dried under nitrogen and the residue was dissolved in 1 ml methanol/dichloromethane (1:1, v/v). Supernanant was then ready for HPLC injection. Extracts were injected into the Shimatzu (Kyoto, Japan) Prominence full HPLC system (Sil-20A Autosampler; LC-20AD solvent delivery system; RF-10 AXL Spectrofluorometeric detector, CBM-20 Alite system controller; Cto-100ASvp column oven) fitted with a Waters Spherisorb type ODS-2, 3 μm C-18 reverse phase HPLC column (150 × 4.6 mm; Phase Separations, UK). Fluorescence detection of vitamin E used excitation at 295 nm and emission at 330 nm and retinol was detected at excitation 330 nm and at emmission 480 nm. Standard solutions of α-tocopherol, γ-tocopherol and retinol in methanol were used for instrument calibration. Chromatography was performed using a mobile phase of ethanol/water (97:3, v/v) at flow rate of 1.05 ml min^-1^. Peaks were identified by comparison with the retention time of standards of tocopherols and retinol (Sigma, Poole, UK). Vitamin E was calculated as the summed concentrations of α-, γ- and δ-tocopherol.

Concentrations of carotenoids were determined using the same HPLC system with a diode array detector at 444 nm, fitted with a Waters Spherisorb type NH2 5 μm 4.6 × 250 mm column (Phase Separations) with a mobile phase of methanol-distilled water (97:3), at flow rate of 1.5 ml min^-1^) as described by Hõrak et al. [86]. The HPLC was calibrated using lutein standards (DSM Nutritional Products Ltd., Basel, Switzerland). Carotenoids were determined from the same extracts using the same HPLC system, but fitted with a Spherisorb S3 ODS2, 5 μm C18 reverse phase column, 250 × 4.6 mm (Phase Separations). Chromatography was performed using two mobile phases: acetonitrile/methanol (85:15 v/v) for 11 min, then gradient change to the second mobile phase (acetonitrile/dichloromethane/methanol, 70:20:10, v/v/v) in a gradient elution at a flow rate of 2 ml min^-1 ^with a diode array detector at 444 nm as described by Granado et al. [87]; total HPLC run time was 24 min per sample. Peaks were identified by comparison with the retention times of a range of carotenoid standards (variously obtained from DSM Nutritional Products Ltd. and Carotenature, Basel, Switzerland), as well as using coelution of individual peaks with known standards.

### Assay of yolk hormones

Egg yolks were homogenized and 50-100 mg of the homogenate was diluted in 1 ml of deionized H_2_O for the analyses. Yolk testosterone, androstenedione, dihydrotestosterone, estradiol, and corticosterone were separated using celite column chromatography according to methods described by Schwabl [17]. Testosterone and corticosterone were quantified using a standard competitive binding radioimmunoassay, using two specific antibodies (testosterone, Esoterix T3-125; corticosterone, Esoterix B3-163; Esoterix, Calabasas Hills, CA, USA) according to methods outlined in Wingfield and Farner [88]. Though a previous study identified several problems related to corticosterone assay of yolk homogenates [89], the combination of highly specific antibody (cross-reactivity with progesterone and a number of other hormones and metabolites closely related in structure is <1%) and chromatographic separation should ensure accurate dosage. Androstenedione, dihydrotestosterone, and estradiol were quantified using commercial I^125^-labeled kits from Diagnostic Systems Laboratories (Webster, TX, USA) (DSL-3800; DSL-9600; DSL-43100, respectively). Inter-assay variation was 6.85% for testosterone, 9.98% for androstenedione, 11.50% for estradiol, and 1.05% for corticosterone. Dihydrotestosterone measurements were only conducted for a subset of samples and were completed in a single assay. Intra-assay variation was 6.54% for testosterone, 4.47% for androstenedione, 6.80% for dihydrotestosterone, 4.27% for estradiol, and 4.82% for corticosterone. Assay lower detection limits were 20 pg/ml for both testosterone and corticosterone, 30 pg/ml for androstenedione, 4 pg/ml for dihydrotestosterone, and 11 pg/ml for estradiol. Average recoveries were 63% for testosterone, 75% for androstenedione, 57% for dihydrotestosterone, 85% for estradiol, and 50% for corticosterone.

Because we expected androstenedione and dihydrotestosterone to be positively correlated with testosterone levels, we only assayed these hormones in a subsample of 61 incubated eggs for androstenedione and 27 incubated eggs for dihydrotestosterone to investigate the correlation with testosterone levels. Testosterone levels were indeed positively correlated with both androstenedione (*r *= 0.53, *P *< 0.001, *n *= 61) and dihydrotestosterone (*r *= 0.47, *P *= 0.013, *n *= 27) (testosterone and androstenedione values were log_10_-transformed, see "Statistical analyses"). Results were qualitatively unchanged if the covariation was tested by a mixed model with clutch identity as a random factor, androstenedione or dihydrotestosterone as dependent variables and testosterone as a predictor (details not shown). Moreover, the positive relationships between testosterone levels and androstenedione or dihydrotestosterone levels did not differ according to laying order (mixed model with androstenedione or dihydrotestosterone as dependent variables and testosterone, laying order and their interaction as predictors; effects of the testosterone × laying order interactions; both *P *> 0.14, details not shown for brevity). In most analyses, for simplicity, we therefore considered only testosterone among the androgens, although we presented descriptive statistics also for androstenedione and dihydrotestosterone.

Since previous studies suggested that hormone levels may change even at the initial stages of incubation [e.g. [61]], we checked whether this was the case by comparing mean hormone levels (testosterone, estradiol, corticosterone) of the sample of 15 unincubated eggs (5 clutches; see "General methods and genetic sex identification") dissected immediately after collection with the mean value of incubated and embryonated eggs (90 eggs; sample sizes may vary slightly because of missing data). The comparison was performed by means of mixed models with clutch identity as a random effect, a two-level fixed factor (incubated or not) and laying order as predictors. The interaction between laying order and incubation status was also included in initial models. Levels of the three hormones did not differ between incubated and freshly laid eggs (all *P *> 0.27) [see also [83]]. The interaction between incubation status and laying order was not significant and was removed from all models (all *P *> 0.29). Hence, our findings concerning yolk hormones should not be confounded by egg incubation taking place before collection [see also [19]].

### Statistical analyses

To limit the leverage of a few high values on regression parameters and improve normality, several variables (cis-lutein, zeaxanthin, dehydrolutein, beta-cryptoxanthin, testosterone, androstenedione, estradiol, corticosterone) were log_10 _or log_10_(x + 1) transformed. We adopted a conservative criterion, and subjected to log-transformation all the variables that resulted non-normal at the Kolmogorov-Smirnov test with α-level set at 0.1. Descriptive statistics were reported in the original scale, where necessary.

We first analysed whether different egg components varied in relation to sex, laying order, and their interaction, in linear mixed models where clutch identity was included as a random factor, to account for non-independence of eggs from the same clutch. These models were re-run on the subset of eggs for which we recorded embryo size (see above), by also including embryo size and its two- and three-ways interaction terms with sex and laying order as fixed effects.

To investigate variation in egg components among clutches, we ran linear mixed models with clutch identity as a random effect and total clutch mass as a predictor. We also included laying date of the first egg and the number of males as clutch-level covariates, to analyse seasonal variation in egg resource allocation [11,13], as well as variation in egg components in relation to sex composition of the clutch [49].

We then investigated the covariation between main egg components (lutein, total carotenoids, vitamin A, vitamin E, testosterone, estradiol, corticosterone) at the egg and clutch level, by adopting the multiple response mixed model approach advocated by Hadfield et al. [90], while concomitantly controlling for the confounding effects of sex, laying order and their interaction, as well as by non-independence of eggs from the same clutch, by including clutch identity as a random factor. This analysis allows estimating correlation coefficients between dependent variables (the concentrations of different egg components in this case) at two levels of variation, among- and within-subjects (clutches in this case). Significance of correlation coefficients for each pair of egg components were obtained by assuming a conservative sample size of 30 [equal to the number of nests; 90]. Dependent variables were standardized before being included in mixed models [90,91]. Differences in correlation coefficients were tested by the *z *statistic [92, see 93 for details of procedure]. The same analyses were re-run for eggs of each sex separately, to investigate sex differences in covariation among egg components. Also in this case, comparisons were made by assuming a conservative sample size of 27 (males) and 23 (females), i.e. the number of clutches that contained at least a male or a female embryo, respectively.

Linear mixed models were run by means of SAS (vers. 9.1.3) PROC MIXED [94], and degrees of freedom were estimated using the Satterthwaite's method, whereas multiple response mixed models were run using the *MCMCglmm *library [91] of software R (vers. 2.11.1) [95]. Additional analyses and details on modelling procedures are reported in the Results section.

## Competing interests

The authors declare that they have no competing interests.

## Authors' contributions

DR and NS conceived the study, collected and analyzed the data, and wrote the paper. MR, MC and RA collected the data, and RA also contributed to statistical data analyses. MC performed molecular sexing of embryos. KJN and FK performed biochemical analyses of egg components. All authors read and approved the final version of the manuscript.
